# The role of genes, intelligence, personality, and social engagement in cognitive performance in Klinefelter syndrome

**DOI:** 10.1002/brb3.645

**Published:** 2017-02-09

**Authors:** Anne Skakkebæk, Philip J. Moore, Anders Degn Pedersen, Anders Bojesen, Maria Krarup Kristensen, Jens Fedder, Peter Laurberg, Jens Michael Hertz, John Rosendahl Østergaard, Mikkel Wallentin, Claus Højbjerg Gravholt

**Affiliations:** ^1^ Department of Endocrinology and Internal Medicine (MEA)Aarhus University HospitalAarhusDenmark; ^2^ Department of Clinical GeneticsAarhus University HospitalAarhusDenmark; ^3^ Department of PsychologyThe George Washington UniversityWashingtonDCUSA; ^4^ Department of Psychology and Behavioral SciencesAarhus UniversityAarhusDenmark; ^5^ Department of Clinical GeneticsSygehus LillebaeltVejleDenmark; ^6^ Department of Mental HealthOdense University ClinicOdenseDenmark; ^7^ Fertility ClinicDepartment of Gynecology and ObstetricsOdense University HospitalOdenseDenmark; ^8^ Department of EndocrinologyAalborg University HospitalAalborgDenmark; ^9^ Department of Clinical GeneticsOdense University HospitalOdenseDenmark; ^10^ Department of PediatricsCentre for Rare DiseasesAarhus University HospitalAarhusDenmark; ^11^ Center for SemioticsAarhus UniversityAarhusDenmark; ^12^ Center of Functionally Integrative NeuroscienceAarhus University HospitalAarhusDenmark; ^13^ Department of Molecular MedicineAarhus University HospitalAarhusDenmark

**Keywords:** cognitive performance, genetics, Klinefelter syndrome, personality, social engagement

## Abstract

**Introduction:**

The determinants of cognitive deficits among individuals with Klinefelter syndrome (KS) are not well understood. This study was conducted to assess the impact of general intelligence, personality, and social engagement on cognitive performance among patients with KS and a group of controls matched for age and years of education.

**Methods:**

Sixty‐nine patients with KS and 69 controls were assessed in terms of IQ, NEO personality inventory, the Autism Spectrum Quotient (AQ) scale, and measures of cognitive performance reflecting working memory and executive function.

**Results:**

Patients with KS performed more poorly on memory and executive‐function tasks. Patients with KS also exhibited greater neuroticism and less extraversion, openness, and conscientiousness than controls. Memory deficits among patients with KS were associated with lower intelligence, while diminished executive functioning was mediated by both lower intelligence and less social engagement.

**Conclusion:**

Our results suggest that among patients with KS, memory deficits are principally a function of lower general intelligence, while executive‐function deficits are associated with both lower intelligence and poorer social skills. This suggests a potential influence of social engagement on executive cognitive functioning (and/or vice‐versa) among individuals with KS, and perhaps those with other genetic disorders. Future longitudinal research would be important to further clarify this and other issues discussed in this research.

## 1 Introduction

Klinefelter syndrome (KS) is the most common sex chromosome disorder, present in 150 of every 100,000 men (Bojesen, Juul, & Gravholt, 2003; Nielsen & Wohlert, 1990). Caused by an additional X chromosome (47,XXY), KS is characterized by hypogonadism, and is associated with increased morbidity because of metabolic, endocrine, circulatory, respiratory, and digestive diseases (Bojesen, Juul, Birkebaek, & Gravholt, 2006). The majority of individuals with KS also exhibit some degree of cognitive deficits, including impaired memory (Fales et al., 2003; Geschwind et al., 1998) and executive function (Geschwind, Boone, Miller, & Swerdloff, 2000; Kompus et al., 2011; Lee et al., 2011), which may have profound, adverse effects on mental and physical health, by increasing psychological stress and health‐risk behaviors as has been found in the general population (Hall, Carroll, & Moore, 2010; Meyer, Springer, & Altice, 2011). Understanding the factors that determine cognitive functioning among patients with KS is important, both for identifying individuals at greater risk for certain cognitive deficits, suggesting the benefit of targeting these patients for treatment, and for developing interventions to address these deficits and their deleterious effects.

In addition to providing valuable information about the interaction between KS and social experience, KS‐related deficits can also inform a number of fundamental questions about cognitive performance: First, is cognitive performance simply a reflection of intelligence? Mean scores for groups of research patients with KS lie below normative averages on tests of general intelligence (Fales et al., 2003; van Rijn, Swaab, Aleman, & Kahn, 2008), and lower intelligence has been associated with poorer working memory and executive function in many studies in the general population (Ackerman, Beier, & Boyle, 2005; Ardila, Pineda, & Rosselli, 2000; Fales et al., 2003; Polderman et al., 2009; Salthouse & Pink, 2008), although these findings are not universal (Friedman et al., 2006).

Second, what role does personality play in cognitive performance? KS is associated with higher levels of neuroticism and lower levels of extraversion, conscientiousness, and openness to experience (Skakkebaek et al., 2013), and these personality characteristics are often correlated with measures of cognitive performance. For example, more neuroticism and less extraversion and conscientiousness are related to poorer working memory (Heffernan & Ling, 2001; Studer‐Luethi, Jaeggi, Buschkuehl, & Perrig, 2012), while more agreeableness and openness to experience have been associated with better executive functioning in the general population (DeYoung, Peterson, & Higgins, 2005; von Hippel, 2007; Kochanska, Aksan, Penney, & Doobay, 2007).

Finally, how does social engagement influence cognitive performance? Like personality, social engagement is a broad construct, comprising diverse elements, such as interpersonal attention, communication, imagination, and general social skills and experience. In some studies, individuals with KS have been shown to exhibit significant deficits in each of these areas of social engagement (van Rijn et al., 2008; Skakkebaek et al., 2013), which are in turn associated with poorer performance on tasks involving working memory and executive function, both in young adults (Seeman et al., 2011) and among the elderly (Bassuk, Glass, & Berkman, 1999; Krueger et al., 2009).

While many studies have examined important bivariate relationships between these factors and KS status, research has yet to examine the simultaneous effects of genetic, personality, and social variables among individuals with and without KS, or how these factors combine to determine differences in cognitive functioning. To address these issues, the current research investigated the impact of intelligence, personality traits, and social engagement on cognitive performance in a sample of patients with KS and in a group of controls matched for age and years of education.

## 2 Materials and Methods

### 2.1 Participants

Participants included 69 patients with KS who ranged in age from 18.1 to 59.8 years old, with an average of 36.4 years. All but two patients with KS were of Danish heritage; the others were of Icelandic and Swedish descent. All patients had the standard 47,XXY KS karyotype. Forty eight (70%) of the patients with KS were receiving testosterone treatment at the time of participation, and 14 of the 21 patients with KS had never been treated with testosterone, while 6 had received testosterone therapy in the past (ranging from 6 months to 7.3 years, with an average of 32.5 months), and 1 patient with KS reported receiving testosterone for an unspecified period in the past.

The 69 controls (all men) were matched for age and years of education. The controls ranged in age from 19.4 to 59.1 years old, with an average of 36.4 years. Although these controls were not karyotyped, and none of them exhibited any characteristics associated with Klinefelter syndrome, and all of them had normal testosterone levels. All controls were of Danish heritage. All participants provided informed consent, and the study was reviewed and approved by The Danish Data Protection Agency and the local ethics committee (Region Midtjylland, Denmark number M‐20080238). This research has also been registered at ClinicalTrials.gov (Clinical trial NCT00999310). Certain genetic, anatomical, and neuropsychological data from this research has been presented previously to address separate research questions (Skakkebaek et al., 2013, 2014).

### 2.2 Procedure

Participants with a verified KS genotype were recruited from endocrinology, genetics, and fertility clinics throughout Denmark, while controls were recruited through advertisements in local hospitals, newspapers, fire departments, and other civil service offices. Inclusion criteria required that participants were between the ages of 18 and 60, and those with a history of neurological disease, serious head injury, color blindness, or substance abuse were excluded from the study. After providing informed consent, participants completed questionnaires to assess personality and social engagement. One week after completing these questionnaires, participants completed a battery of standardized cognitive tests to assess intelligence, working memory, and executive function. These tests were administered in the same order to all participants by trained research assistants, under the supervision of a senior specialist in clinical neuropsychology.

### 2.3 Measures

#### 2.3.1 Personality

Participants’ personality traits were assessed using Revised NEO Personality Inventory (NEO PI‐R), short form (Costa & McCrae, 1992), which includes measures of neuroticism (12 items), extraversion (12 items), agreeableness (12 items), conscientiousness (12 items), and openness to experience (12 items). *Neuroticism* reflects to an individual's tendency to experience negative emotion (e.g., “I am easily bothered by things”); *extraversion* is characterized by pronounced engagement with the external world (e.g., “I don't mind being the center of attention”); *agreeableness* refers to an individual's desire for social harmony, and to get along with others (e.g., “I am interested in people”); *conscientiousness* is a tendency to show self‐discipline, act dutifully, and strive for achievement (e.g., “I am exacting in my work”); and *openness to experience* reflects one's interest in, and appreciation for art, emotion, curiosity, novelty, adventure, and a variety of experiences (e.g., “I spend time reflecting on things”). For all items, participants indicated how well each statement described them—relative to other people of the same sex and comparable age—on a scale from 1 (“very inaccurate”) to 5 (“very accurate”). Items within each trait were then combined to create an overall score for each personality type (McCrae & John, 1992). The reliability of aggregate measures such as NEO PI‐R can be assessed using Cronbach′s alpha, with values closer to 1 indicating greater internal consistency. The current measures were quite reliable, with Cronbach's alpha's ranging from 0.70 to 0.90.

#### 2.3.2 Social engagement

Participants’ level of social engagement was assessed using the Autism Spectrum Quotient (AQ), which includes measures of attention to detail (10 items), attention switching (10 items), imagination (10 items), communication (10 items), and general social skills (10 items) (Baron‐Cohen, Wheelwright, Skinner, Martin, & Clubley, 2001). *Attention to detail* refers to an individual's tendency to focus on small details in his or her immediate environment (e.g. “I tend to notice details that others do not”); *attention switching* reflects an individual's ability to transfer attentional focus from one target to another (e.g. “I frequently get so strongly absorbed in one thing that I lose sight of other things”); *imagination* refers to an individual's capacity for abstraction and envisioning alternative realities (e.g. “I find it very easy to play games with children that involve pretending”); *communication* reflects one's ability to relate his or her thoughts, feelings, and ideas to others (e.g. “I find making up stories easy”); and social skills include interests and abilities that facilitate positive social interactions and relationships (e.g. “I find social situations easy”). For all items, participants indicated how well each statement described them on a scale from 1 (“definitely disagree”) to 4 (“definitely agree”). Negatively worded items were reverse coded, so that higher scores for each item reflected more of each social characteristic. Items within each measure were then combined to create an overall score for each aspect of social engagement. Cronbach's alphas in the current study for these social engagement measures range from 0.61 to 0.74.

#### 2.3.3 Testosterone

Testosterone levels for all participants were measured by liquid chromatography tandem mass spectrometry using Perkin Elmer′s CHS steroid MS kit. The lower limit of detection was 0.1 nmol/L and the working range 0.2–100 nmol/L.

#### 2.3.4 Cognitive functioning

Cognitive functioning in this research included a general measure of intelligence and two specific measures of cognitive performance: working memory and executive function. *Intelligence* was assessed in terms of a full‐scale intelligence quotient (FSIQ), which combined two subscales of verbal IQ—vocabulary (V) and similarities (S)—and two subscales of performance IQ—matrix reasoning (MR) and block design (BD)—from the Wechsler Adult Intelligence Scale, Third Edition (WAIS‐III) (Wechsler, 1997). In accordance with the WAIS‐III Danish reference material, each participant's FSIQ was determined using the following regression equation FSIQ = 40.21 + (1.13 × S) + (1.38 × MR) + (2.10 × V) + (1.35 × BD). *Working memory* was assessed by combining two memory subscales from the WAIS‐III—digit span (DS) and letter‐number sequencing (LN)—with the Rey Auditory Verbal Learning Test (RAVL) (Nielsen, Knudsen, & Daugbjerg, 1989). These three measures were standardized and combined, and the sum was then divided by 3 to create an aggregate working memory score for each participant.

*Executive function* was assessed using the Wisconsin Card Sorting Test (WCST), which is designed to measure an individual's cognitive flexibility in the face of changing schedules of reinforcement (Berg, 1948). In the WCST, participants are repeatedly presented with sets of stimulus cards on a computer screen that they are told to match, but not how. Participants are subsequently told whether each match is right or wrong, and trials are continued until a certain number of cards are matched correctly. The three outcomes from the WCST considered in this research included the number of trials, the percentage of errors, and the percentage of perseverative responses—all of which are considered negative indices of executive function (Monchi, Petrides, Petre, Worsley, & Dagher, 2001). These three measures were standardized and combined, and the sum was then divided by 3 to create an aggregate executive‐function score for each participant. Cronbach's alphas for these three aspects of cognitive functioning ranged from 0.76 to 0.93.

### 2.4 Analysis

Initial analyses were conducted comparing patients with KS with control participants in terms of age, testosterone, personality, social engagement, and cognitive functioning. This included comparative frequency distributions among patients with KS and controls for age, testosterone, and each measure of personality and social engagement, as well as mean/median comparisons between patients with KS and controls for all dependent measures. Correlations were then calculated between each of the personality, social engagement, and cognitive measures associated with participants’ KS status, age, and testosterone levels.

Four sets of regression analyses were conducted for both aspects of cognitive performance (working memory and executive function) in which each of these two dependent variables – after entering KS status – was regressed on following independent variables (1) personality traits, (2) social engagement measures, (3) intelligence, and (4) testosterone status, respectively. Finally, two path analyses—one for working memory and one for executive function—were conducted to identify the unique associations between each of the bivariate predictors of cognitive performance.

Continuous measures were tested for normality using the Kolmogorov‐Smirnov test, and those with distributions of Dα > 0.23 were considered non‐normal, and were normalized using a log‐linear (ln+1) transformation. Differences between patients with KS and controls measures were assessed using two‐sample Student's *t*‐tests, with *p*‐values lower than .05 considered significant. All analyses were conducted using SPSS version 19.0 (SPSS Inc., Chicago, IL, USA).

## 3 Results

### 3.1 Patients with KS vs. Controls

#### 3.1.1 Personality

**Figure 1:**
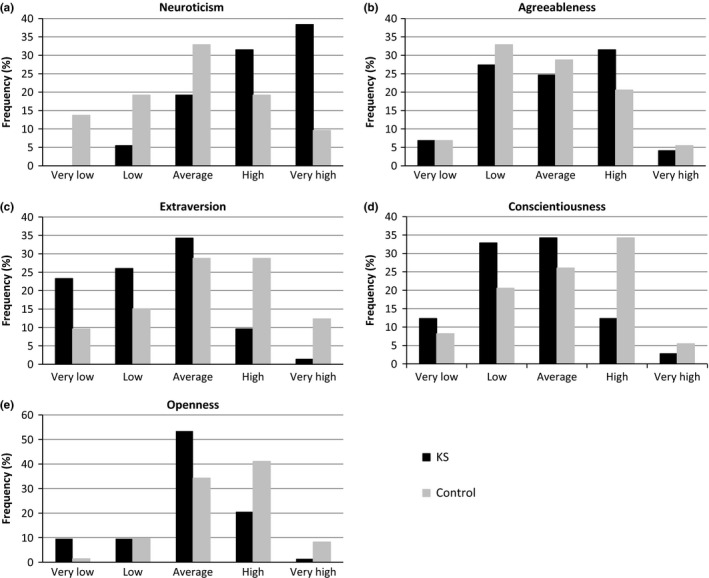
Frequency distributions of scores on the five personality dimensions in patients with Klinefelter syndrome and controls. This figure has previously been presented in a recent review on Klinefelter syndrome (Skakkebaek et al., 2015)

Neuroticism, extraversion, and agreeableness were all normally distributed among control participants. However, the percentage of patients with KS increased monotonically with increasing neuroticism, the percentage of patients with KS decreased as extraversion increased, but was normally distributed across agreeableness (see Figure 1). Controls were skewed slightly toward more openness, and even more so toward greater conscientiousness, while patients with KS were normally distributed across openness, and skewed toward less conscientiousness. Relative to controls, patients with KS expressed significantly more neuroticism, less extraversion, conscientiousness, and openness to experience (*p*s ≤ .01), but the two groups did not differ significantly in terms of agreeableness (*p *> .33) (see Table 1). These personality data have been presented previously in the summary form in a recent review on Klinefelter syndrome (Skakkebaek, Wallentin, & Gravholt, 2015).

**Table 1:** Age, testosterone, and psychological characteristics of patients with Klinefelter syndrome and controls

*N*	KS	Controls	*p*‐value	Cohen's d
	69	69		
	Mean/Median (SD)	Mean/Median (SD)		
Age	36.4/35.6 (9.88)	36.4/35.9 (9.58)	.97^b^	0.00
Testosterone (nmol/L)	17.4/15.6 (11.2)	14.2/13.3 (5.82)	.04^b^	0.36
Personality				
Neuroticism	4.13/4.16 (0.15)^a^	3.90/3.93 (0.24)^a^	<.001^b^	1.15
Extraversion	3.73/3.81 (0.31)^a^	3.95/4.03 (0.29)^a^	<.001^b^	0.73
Openness	3.89/3.95 (0.23)^a^	4.01/4.04 (0.17)^a^	.001^b^	0.60
Agreeableness	3.91/3.95 (0.21)^a^	3.87/3.90 (0.23)^a^	.33^b^	0.18
Conscientiousness	3.81/3.83 (0.25)^a^	3.91/3.95 (0.25)^a^	.01^b^	0.40
Social Engagement				
Attention to detail	1.82/1.95 (0.35)^a^	1.84/1.95 (0.34)^a^	.75^b^	0.06
Attention switching	1.78/1.95 (0.45)^a^	2.01/2.08 (0.33)^a^	.001^b^	0.58
Imagination	1.82/1.95 (0.39)^a^	2.04/2.08 (0.28)^a^	<.001^b^	0.65
Communication skills	2.05/2.08 ± (0.34)^a^	2.17/2.20 (0.22)^a^	0.01^b^	0.42
Social skills	1.95/2.01 (0.41)^a^	2.14/2.30 (0.31)^a^	0.003^b^	0.52

NEO PI‐R, Revised NEO Personality Inventory, short form; AQ, Autism Spectrum Quotient.

a Ln+1 transformed data.

b Student's *t*‐test.

#### 3.1.2 Social engagement

**Figure 2:**
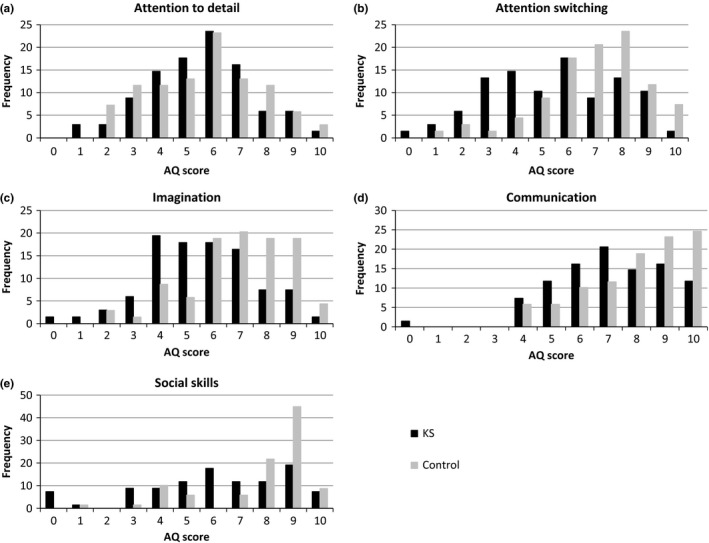
Frequency distributions of scores on the Autism Spectrum Quotient (AQ) subscales in patients with Klinefelter syndrome and controls

As shown in Figure 2, controls scored higher on attention switching, imagination, communication, and social skills, while the scores of patients with KS were more evenly distributed across these scales. While attention‐to‐detail scores were comparably – and normally – distributed for both patients with KS and controls (*p *> .75), patients with KS scored significantly lower than controls on attention switching, imagination, communication, and social skills (*p*s < .01)(see Table 1).

#### 3.1.3 Cognitive functioning

General intelligence scores of patients with KS were significantly lower than those of the controls (*p *< .001)(see Table 2). Patients with KS also performed more poorly on all measures of both working memory and executive function (*p*s ≤ .001).

**Table 2:** Neurocognitive ability among patients with Klinefelter syndrome and controls

*N*	KS	Controls	*p*‐value	Cohen′s d
	69	69		
	Mean/median (SD)	Mean/median (SD)		
Intelligence	87.3/88.2 (12.4)	102.9/103.2 (11.6)	<.001^b^	1.30
Working memory				
WAIS‐III DS	2.60/2.56 (0.21)^a^	2.75/2.77 (0.17)^a^	<.001^b^	0.79
WAIS‐III LN	2.27/2.30 (0.33)^a^	2.44/2.48 (0.20)^a^	.001^b^	0.62
RAVL total	43 (21–66)	50 (29–70)	0.27	<0.001
	3.75/3.78 (0.23)^a^	3.93/3.95 (0.20)^a^	<.001^b^	0.84
Overall working memory score	−0.32/−0.42 (0.84)	0.33/0.28 (0.67)	<.001^b^	0.86
Executive function				
WCST cards	4.68/4.78 (0.20)^a^	4.52/4.44 (0.20)^a^	<.001^b^	0.80
WCST errors (%)	3.32/3.30 (0.47)^a^	3.07/2.94 (0.45)^a^	.001^b^	0.54
WCST persev.responses (%)	2.67/2.64 (0.44)^a^	2.40/2.30 (0.41)^a^	<.001^b^	0.63
Overall executive‐function score	0.27/0.33 (0.92)	−0.31/−0.67 (0.85)	<.001^b^	0.65

IQ, intelligence quotient; WAIS‐III, Wechsler Adult Intelligence Scale – third edition; WAIS‐III DS, WAIS‐III Digit Span; WAIS‐III LN, WAIS‐III Letter‐Number Sequencing; RAVL, Rey Auditory Verbal Learning Test; Wisconsin Card Sorting Test.

Data are medians (total range) or means ± SD. Mann‐Whitney test rank‐sum test.

*t*‐test.

a ln+1 transformed data.

b Student's *t*‐test.

### 3.2 Modeling cognitive performance

#### 3.2.1 Testosterone

As shown in Table 1, patients with KS had a significantly higher levels of testosterone, reflecting the fact that patients with KS receiving treatment with testosterone were receiving a high doses of exogenous testosterone (*p *< .05). Testosterone levels among patients with KS receiving testosterone therapy (20.7 nmol/L ± 11.4) were significantly higher than those not receiving this therapy (9.61 nmol/L ± 5.38) (*p *< .001).

#### 3.2.2 Bivariate predictors

Because patients with KS and controls did not differ significantly in terms of age, agreeableness, or attention to detail (*p*s ≥ .33), and because neither conscientiousness, attention switching nor testosterone levels were associated with cognitive performance, these variables were not included in subsequent analyses. As shown in Table 3, higher intelligence scores were associated with better working memory and executive‐function performance (*p*s < .01). Better memory and executive function were also associated with lower neuroticism, and more extraversion and openness to experience (*p*s < .05). Greater imagination, communication, and social skills were all related to better working memory (*p*s < .05), but only imagination and social skills predicted executive‐function performance (*p*s < .05).

**Table 3:** Correlations between memory and executive functions and testosterone, IQ, personality traits and social‐engagement skills

	Testosterone	IQ	Personality			Social engagement		
			Neuroticism	Extraversion	Openness	Communication	Social skills	Imagination
Social engagement								
Communication	−0.145	0.224*	−0.407**	0.481**	0.405**			
Social skills	−0.125	0.106	−0.504**	0.714**	0.336**			
Imagination	−0.083	0.409**	−0.169*	0.321**	0.421**			
Overall score	−0.150	0.186*	−0.494**	0.657**	0.400**			
Working memory								
WAIS‐III DS	0.042	0.596**	−0.163	0.176*	0.182*	0.140	0.171*	0.202*
WAIS‐III LN	−0.083	0.524**	−0.134	0.184*	0.231**	0.192*	0.149	0.208*
RAVL total	0.001	0.441**	−0.135	0.259**	0.306**	0.186*	0.242**	0.427**
Overall score	−0.005	0.636**	−0.174*	0.244**	0.270**	0.187*	0.228**	0.338**
Executive function								
WCST cards	0.050	−0.466**	0.186*	−0.200*	−0.239**	−0.126	−0.331**	−0.226**
WCST errors %	0.031	−0.415**	0.180*	−0.174*	−0.182*	−0.123	−0.329**	−0.201*
WCST response %	−0.007	−0.396**	0.168*	−0.172*	−0.155	−0.121	−0.320**	−0.203*
Overall score	0.017	−0.444**	0.175*	−0.194*	−0.203*	−0.120	−0.348**	−0.228**

*p < .05; **p < .01.

#### 3.2.3 Multiple regressions

When KS status and personality measures (i.e., neuroticism, extraversion, and openness to experience) were combined to predict working memory (dependent variable), the overall model was significant (*R* ^2^ = .19, *p *< .001), but KS status was the only significant predictor (*p *< .001) (see Table 4). When working memory was regressed on KS status and social engagement (i.e., imagination, communication, and social skills), the full model was again significant (*R* ^2^ = .24, *p *< .001), with both KS status and imagination emerging as significant predictors (*p*s < 0.01). Combining KS status and intelligence also predicted working memory (*R* ^2^ = .60, *p *< .001), but this was attributable to the impact of intelligence (*p *< .001). Combining KS status and testosterone status, the full model was again significant (*R* ^2^ = .17, *p *< .001), but this was attributable to the impact of KS status.

**Table 4:** Regression models predicting memory performance

Independent variables	Standardized β coefficient	t‐score	*p*‐value
Personality			
KS Status	−0.35	−3.74	<.001
Neuroticism	0.06	0.57	.57
Extraversion	0.08	0.84	.40
Openness	0.14	1.54	.13
Overall model		*F*_4,135_ = 7.50, *p *< .001, *R* ^2^ = 0.19	
Social engagement			
KS Status	−0.29	−3.50	<.01
Social skills	0.07	0.69	.49
Communication	−0.01	−0.12	.91
Imagination	0.28	3.23	<.01
Overall model		*F*_4,133_ = 10.18, *p *< .001, *R* ^2^ = 0.24	
Intelligence			
KS Status	0.02	0.34	.74
Intelligence	0.79	12.2	<.001
Overall model		*F*_2,135_ = 100.8, *p *< .001, *R* ^2^ = 0.60	
Testosterone			
KS Status	−0.46	−5.26	<.001
Testosterone status	−0.15	−1.70	.09
Overall model		*F*_2,135_ = 14.0, *p *< .001, *R* ^2^ = 0.17	

As shown in Table 5, KS status and personality measures (i.e., neuroticism, extraversion, and openness to experience) combined to significantly predict executive function (dependent variable) (*R* ^2^ = .11, *p *< .05), with KS status again emerging as the lone significant predictor (*p *< 0.01). Executive function was also predicted by KS status and social engagement (i.e., imagination, communication, and social skills) (*R* ^2^ = .17, *p *< .001), with KS status and social skills as the significant predictors (*p*s ≤ 0.01. KS status and intelligence combined to significantly predict executive function (*R* ^2^ = .15, *p *< .001), which was principally attributable to the impact of intelligence (*p *≤ .01), although KS status was a marginal predictor (*p *< .06). When combining KS status and testosterone status, the overall model was significant (*R* ^2^ = .10, *p *< .001), but this was attributable specifically to KS status.

**Table 5:** Regression models predicting executive function

Independent variables	Standardized β coefficient	t‐score	*p*‐value
Personality			
KS Status	0.28	2.82	<0.01
Neuroticism	0.00	0.01	0.99
Extraversion	−0.05	−0.43	0.67
Openness	−0.08	−0.88	0.38
Overall model		*F*_4,137_ = 4.14, *p* = .003, *R* ^2^ = 0.11	
Social engagement			
KS Status	0.23	2.60	0.01
Social skills	0.29	2.98	<0.01
Communication	−0.16	−1.65	0.10
Imagination	0.12	1.41	0.16
Overall model		*F*_4,135_ = 6.65, *p *< .001, *R* = 0.17	
Intelligence			
KS Status	0.18	1.87	0.06
Intelligence	−0.26	−2.78	<0.01
Overall model		*F*_2,137_ = 11.8, *p *< .001, *R* ^2^ = 0.15	
Testosterone			
KS Status	0.308	3.42	0.001
Testosterone status	−0.02	−0.23	0.82
Overall model		*F*_2,137_ = 7.57.0, *p *< .001., *R* ^2^=0.10	

#### 3.2.4 Path models

**Figure 3:**
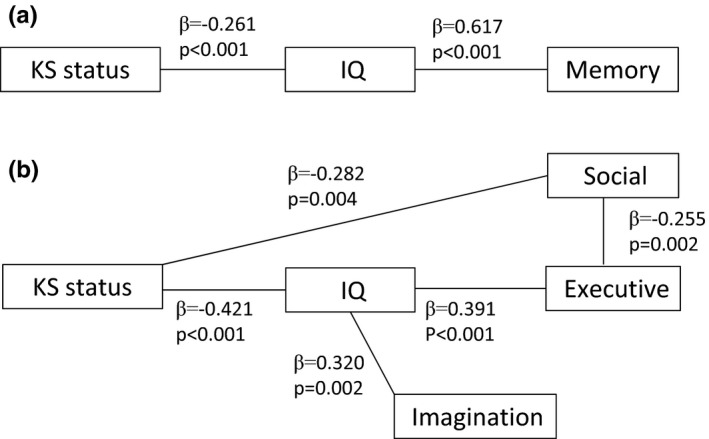
Path models of memory performance (a) and executive performance (b) with β standard coefficients and *p*‐values

In the first path model (Figure 3a), intelligence fully mediated the effect of KS status on memory performance, indicating that poorer working memory among KS patients was attributable directly to differences in intelligence (*p*s < .001). In the second path model (Figure 3b), intelligence partially mediated the influence of KS status on executive function, indicating that poorer executive function among patients with KS was attributable, at least in part, to differences in intelligence (*p *< .01). In addition, KS also led to poorer social engagement, which was in turn related to executive‐function deficits (*p*s ≤ .002). Finally, intelligence also mediated the impact of KS status on imagination (*p *= .002).

## 4 Discussion

In this research, working memory deficits among patients with KS were attributable directly to lower intelligence (as measured by IQ), while their executive‐function deficits were associated with both lower intelligence and poorer social skills. Consistent with previous findings, linking KS status and intelligence with cognitive performance (Bender, Linden, & Harmon, 2001; Bender, Linden, & Robinson, 1993; Boada, Janusz, Hutaff‐Lee, & Tartaglia, 2009; Kompus et al., 2011; O'Brien & Pearson, 2004; Ross et al., 2008; Walzer, Bashir, & Silbert, 1990), this research represents a relatively rare biopsychosocial examination of cognitive performance among individuals with chromosomal abnormalities and, to the best of our knowledge, the first such examination among patients with KS. These results also help explain cognitive deficits among patients with KS by identifying the central—though not necessarily exclusive—role of intelligence in this process.

The significant links between KS status, social skills, and executive function present a number of possibilities. First, KS and executive function may simply operate jointly—albeit separately—to affect social skills. Alternatively, KS may operate through social engagement to influence executive function. While the direction of the relationship between KS and social skills is clear, the link between social skills and executive function could be in either direction, or both. In either case, a link between KS, social skills, and executive function makes sense, for while memory is more declarative, executive function requires more abstract conceptual processing to adapt one's behavior to ongoing changes, including the flow of social stimuli. The current findings for the combined (KS and controls) path model are consistent with previous literature linking social skills and executive function in the general population (Kiley‐Brabeck & Sobin, 2006; Muscara, Catroppa, & Anderson, 2008), as well as with research that has found significant, long‐term effects of one's social environment on executive functioning and underlying neuroplasticity in children (Bryck & Fisher, 2012).

Thus, while executive function almost certainly influences social skills, the current results indicates the potential for a converse relationship as well, by which cumulative social perceptions and experiences may mediate the impact of KS on executive function. If so, executive functioning among patients with KS and perhaps others may be improved by enhancing individuals’ social engagement, a notion that is further supported by social‐engagement interventions demonstrating improvements in executive functioning among children with autism spectrum disorders (Stichter, O'Connor, Herzog, Lierheimer, & McGhee, 2012; Tyminski & Moore, 2008). However, future longitudinal research would be necessary to address these questions, including the impact of duration and quality of social interactions, as well as the generalizability of these effects.

Poorer imagination skills among patients with KS were attributable to lower intelligence in the context of executive function. Consistent with previous results linking intelligence and imagination in the general population (Gregory, Nettelbeck, & Wilson, 2010; Schubert, 1973), these findings also suggest that while imagination—an element of creativity—can be considered one aspect of intelligence, intelligence, and imagination also operate as separate, factors, with differential roles in cognitive functioning.

Consistent with prior research, this study found that intelligence was positively and directly related with both memory performance and executive function. Noting that IQ scores can be influenced by genes, education, and various life experiences, Dennis et al. (2009) also posit that memory and executive function are not only related to, but nested within, intelligence. Similar arguments have been made regarding the relationship between KS and certain biological processes (Bojesen, Høst, & Gravholt, 2010). Whatever the degree of overlap between memory, executive function, and intelligence, the current results show that these constructs operate differently, and *independently*, on other factors.

We did not find any link between testosterone level and cognitive performance, nor did testosterone status (treated vs untreated) predict cognitive function. This is consistent with prior research finding. No cognitive differences observed between KS patients treated with testosterone and those who were not (Skakkebaek et al., 2013). In further support of this, no effect of testosterone treatment on cognitive functions is seen in hypogonadal men without KS (Holland, Bandelow, & Hogervorst, 2011). The testosterone levels in this study were measured by a single blood sample at one particular time. Thus, it may not represent the testosterone levels over prolonged periods. Furthermore, it is possible that the impact of testosterone on the cognitive function may occur early in life (Samango‐Sprouse et al., 2015), resulting in a “ ceiling effect” for the subsequent influence of both hypogonadism and testosterone therapy on cognitive functioning. Further prospective, longitudinal and blinded research is needed to address this question.

The current matched‐control design enabled this study to control for the effects of age and education. However, this research did not control for a variety of other factors, including family history, mental, and physical health (except for neurological disease), health‐related behaviors other than substance abuse, and other personal experiences. Further research examining the impact of these and other factors on the cognitive function of patients with KS—and others—would help refine our understanding of these outcomes.

Although this research was cross‐sectional, the directionality of genetic factors such as KS is clear. Similarly, personality traits are established early in life, social‐engagement skills are relatively stable over time, and both are thought to precede situational assessments of cognitive performance. On the other hand, while intelligence may influence imagination, the reverse is also possible. The directionality of these and other relationships can be assessed more directly by future longitudinal research, including tracking of developmental trajectories (e.g., through growth‐curve modeling) of these factors among patients with KS and other populations associated with cognitive impairment, including Down′s syndrome (Roizen & Patterson, 2003), Turner syndrome (Berkovitz, Stamberg, Plotnick, & Lanes, 1983), Prader Willi syndrome (Curfs & Fryns, 1992), and autism spectrum disorders (ASDs) (O'Brien & Pearson, 2004; Stichter et al., 2012).

Given that patients with KS vary widely in their neuropsychological phenotype, the fact, that the patients with KS participating in this study were recruited through fertility clinics, endocrinology clinics and genetic departments may introduce ascertainment bias. Thus, these patients may differ systematically from others with KS who are either not diagnosed or followed by hospital departments and fertility clinics. It is estimated that, only 25% of males with KS are actually diagnosed, which may limit the external validity of KS research to date. However, the patients with KS in the current study exhibited a broad range of intelligence and neuropsychological phenotype, as well as age and testosterone treatment. In addition, the neuropsychological profiles of these patients correspond well to the profiles of patients with KS in the existing literature, supporting the generalizability of the current results to others with KS who have been identified.

This research involved a comprehensive, biopsychosocial study of individuals with a sex chromosomal disorder, and is the first to examine the combined effects of intelligence, personality, and social engagement on cognitive performance among patients with KS and controls. In addition to clarifying the respective roles these factors play in working memory and executive function, these findings also have important implications for strategies to improve cognitive performance among patients with KS, and perhaps in the general population. This interdisciplinary approach can also serve as a model for future research, the results of which can help us better understand and address genetic disorders, as well as the profound personal, social, and economic problems they can cause.

## Conflict of Interest

Authors do not have any potential sources of conflict of Interest.
